# The Interplay Between Diabetes and Oral Health: A Comprehensive Bibliometric Analysis of Clinical Trials (1967-2024)

**DOI:** 10.7759/cureus.58667

**Published:** 2024-04-20

**Authors:** Namrata Dagli, Mainul Haque, Santosh Kumar

**Affiliations:** 1 Research, School of Dentistry, Karnavati Scientific Research Center, Karnavati University, Gandhinagar, IND; 2 Pharmacology and Therapeutics, National Defence University of Malaysia, Kuala Lumpur, MYS; 3 Periodontology and Implantology, School of Dentistry, Karnavati University, Gandhinagar, IND

**Keywords:** chronic metabolic disorder, diabetes mellitus, chronic periodontitis, periodontal therapy, overlay visualization, glycated hemoglobin, oral health, bibliometric review, network visualization, scientometric analysis

## Abstract

Recognizing the complex interaction between diabetes and oral health is crucial, considering the increasing worldwide prevalence of these conditions. This bibliometric analysis delves into the extensive body of literature concerning the impact of diabetes on oral health, utilizing data retrieved from PubMed. The publishing trends indicate a growing research interest in the field over time, with notable peaks and declines. Coauthorship analyses of authors and institutions illuminated collaborative networks within the research community. Two departments at Ahvaz Jundishapur University of Medical Sciences in Iran, namely the Department of Periodontology within the School of Dentistry and the Diabetes Research Center within the Health Research Institute, demonstrated the highest total link strength. The co-occurrence analysis of keywords also unveiled thematic clusters, reflecting research focus areas and evolving trends. The analysis of topic trends highlighted persistent research interests in topics, such as type 2 diabetes mellitus, glycated hemoglobin, periodontitis, and therapy for chronic periodontitis, with shifts in therapeutic modalities investigated. The thematic map suggests that dental implants and tumor necrosis factor-alpha are emerging terms in the field that have gained more traction recently. Furthermore, the analysis of scientific production by country indicated varied contributions, with Brazil leading in publication output. Analysis of collaboration among corresponding authors' countries identified Italy exhibiting substantial international collaboration, while most of the countries primarily produced single-country publications. This comprehensive analysis provides insights into the multifaceted landscape of research on diabetes and oral health, emphasizing ongoing efforts to understand and address the complex interplay between these conditions.

## Introduction and background

Diabetes mellitus and oral health represent two intricate domains of human health that intersect in profound and multifaceted ways. Over the past decades, an increasing body of research has sought to elucidate the intricate relationship between these seemingly disparate yet interconnected fields. Diabetes, a chronic metabolic disorder characterized by hyperglycemia, not only affects systemic health but also manifests significant repercussions on oral health and its associated structures [1-7]. Type 2 diabetes is posing major health challenges and a public health burden in adolescents too [8]. A study demonstrated a high prevalence of periodontitis observed in children and adolescents with type 2 diabetes [9].

Understanding the intricate interplay between diabetes and oral health is imperative, considering the escalating global burden of both conditions. Diabetes affects millions worldwide, with its prevalence steadily rising across diverse populations [10]. Simultaneously, oral diseases, often underestimated in their impact, pose significant public health challenges, exerting substantial economic burdens, and compromising individuals' quality of life [11].

In response to these intertwined health challenges, researchers across various disciplines have investigated the complex associations between diabetes and oral health [1-7,12-14]. Given the expanding literature landscape in this domain, a comprehensive bibliometric analysis presents an invaluable opportunity to systematically map the research trajectory, identify key thematic areas, and discern emerging trends. This bibliometric exploration facilitates a retrospective understanding of past research endeavors and informs future directions for inquiry, policy formulation, and clinical practice. This bibliometric analysis aimed to identify leading authors, their affiliations and coauthorship, keyword frequency over time and cooccurrence, and scientific production of countries and collaborations between them to glean insights into the evolution, current state of knowledge, and future research directions regarding the impact of diabetes on oral health.

## Review

Materials and methods

Database Selection and Search Strategy

The primary database for this bibliometric analysis was PubMed, a comprehensive repository of biomedical literature maintained by the National Library of Medicine (NLM). PubMed offers extensive coverage of peer-reviewed articles, encompassing a wide array of disciplines within the biomedical and life sciences domains.

**Figure 1:**
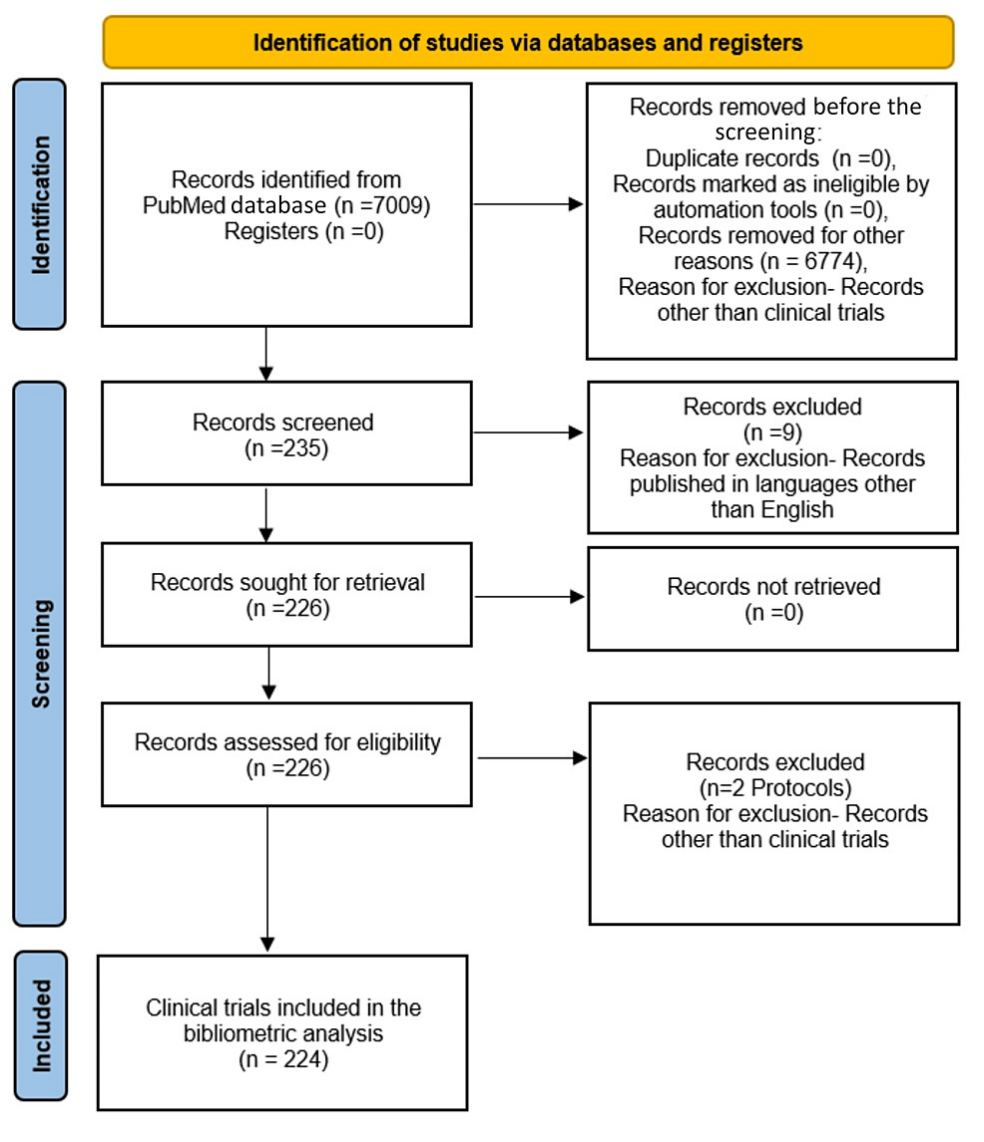
Process of selection of studies.

A systematic search strategy was devised to retrieve relevant literature about the impact of diabetes on oral health. The search was conducted on March 31, 2024, ensuring retrieval of the most up-to-date literature available in the PubMed database up to that date. The search strategy comprised a combination of relevant keywords and Medical Subject Headings (MeSH) terms tailored to capture pertinent studies addressing the intersection of diabetes and oral health. The following search string was employed: ("diabetes" OR "diabetes mellitus") AND ("oral health" OR "dental health" OR "periodontal disease" OR "dental caries" OR "Oral candidiasis" OR "xerostomia" OR "Burning mouth syndrome" OR "dental implant" OR "Periodontitis" OR "Oral ulcer"). This search string aimed to include clinical trials published in English addressing various aspects of the relationship between diabetes and oral health. Articles other than clinical trials were excluded. In addition, irrelevant or non-English language clinical trials were not included in the analysis. The study selection process is depicted in the flow chart generated according to Preferred Reporting Items for Systematic Reviews and Meta-Analyses (PRISMA) guidelines (Figure 1) [15].

Data Extraction and Duplicate Removal

After retrieving pertinent articles from PubMed, we extracted bibliographic data into a text file. This data encompassed publication years, author names, journal titles, article titles, abstracts, and keywords. We ensured the absence of duplicate articles. Subsequently, we manually inspected the extracted bibliographic data for articles other than clinical trials, identifying and eliminating two clinical trial protocols.

Bibliometric Analysis

Bibliometric analysis was performed using the two following software tools: VOSviewer (Leiden, Netherlands: Leiden University) [16] and Biblioshiny (Naples, Italy: Aria and Cuccurullo) [17]. VOSviewer facilitated the visualization of bibliographic data through network visualization techniques, enabling the identification of key clusters, coauthorship, and keyword cooccurrence within the literature. Biblioshiny was used to analyze and visualize the leading contributors, thematic evolution, and the frequency of collaboration between nations across the world. In addition, BioRender (ON, Canada: Science Suite Inc.) and MS Excel (Redmond, WA: Microsoft Corporation) were used to visualize the analysis findings [18].

Results

According to the search conducted on PubMed, there are a total of 7009 results related to the impact of diabetes on oral health. Among these results, 2536 articles were published within the last five years, indicating a significant volume of recent research in this field. The distribution of publication types within the retrieved literature is diverse, encompassing various scholarly contributions. Notably, there are 15 books and documents, including foundational literature. Case reports constitute a substantial portion of the 218 articles that provide insights into individual patient cases. There were 235 articles on clinical trials, with phase 2 trials being the most prevalent among different phases. Comments and editorials contribute to the discourse, with 88 and 28 articles offering critical analyses and perspectives. Reviews dominate the landscape, with 1306 articles reflecting a comprehensive synthesis of existing knowledge. Additionally, there are four protocols delineating research methodologies and one newspaper article providing potentially unique perspectives. Observational studies, numbering 57 articles, contribute essential real-world data to the literature, ensuring a well-rounded understanding of the subject matter. Overall, this distribution underscores the multifaceted approach to research and dissemination within the field. After selecting the English filter, 226 clinical trials remained. After further refinement and removal of two protocols, 224 clinical trials were included in the analysis. The process of selection of studies is depicted in the flow chart (Figure 1).

A total of 1198 authors published the clinical trials in 101 sources between 1967 and 2024. The annual growth rate is 3.19%, and international collaboration is 14.29%. There are four authors with single-authored documents, and coauthors per document are 6.39. A total of 1051 authors' keywords were identified.

Publishing Trends

**Figure 2:**
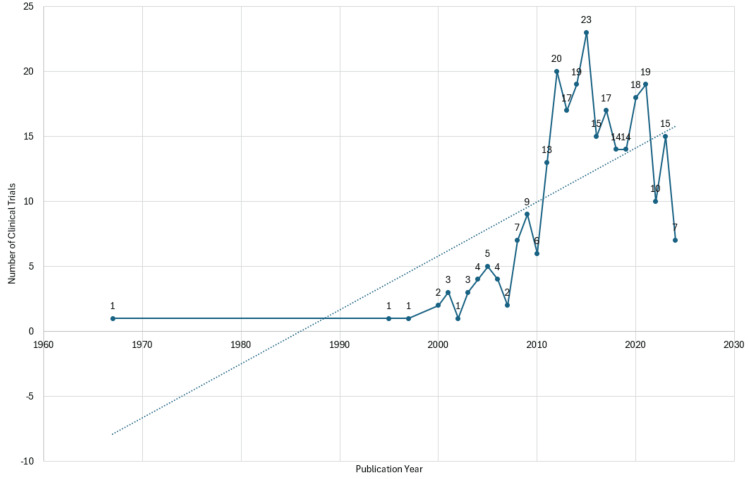
Annual scientific production analysis of clinical trials on the impact of diabetes on oral health. The image is created by the author (Namrata Dagli) of this study.

An erratic publishing pattern of clinical trials concerning the topic is apparent from Figure 2. Short declines follow brief spikes in publications. Nevertheless, the trend line depicted in the graph indicates an overall upward trend in the publication of clinical trials on the subject. The peak number of papers was observed in 2015 in PubMed, while the most significant surge was witnessed between 2010 and 2012. The most substantial decrease occurred between 2021 and 2022, followed by a decline between 2015 and 2016.

*Most Relevant Author*s

**Figure 3:**
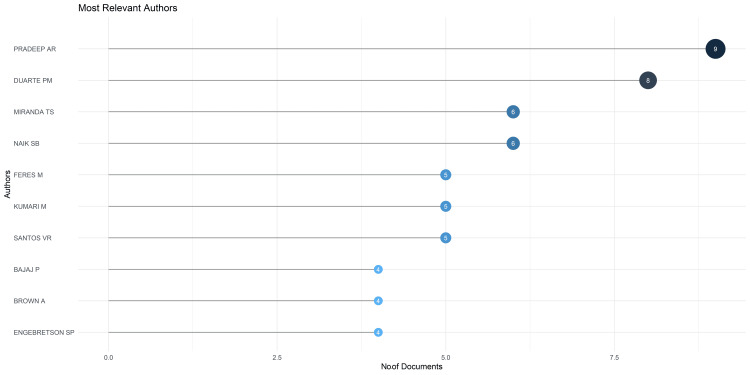
Most relevant authors based on the number of published clinical trials on the impact of diabetes on oral health. The image is created by the author (Namrata Dagli) of this study.

The most prominent author in terms of the number of clinical trials on the topic is Pradeep AR, who has nine publications, closely followed by Duarte PM, who has eight publications. The top 10 most relevant authors accounted for 25% of the total publications analyzed in this study (Figure 3).

Coauthorship Analysis and Overlay Visualization of Authors

**Figure 4:**
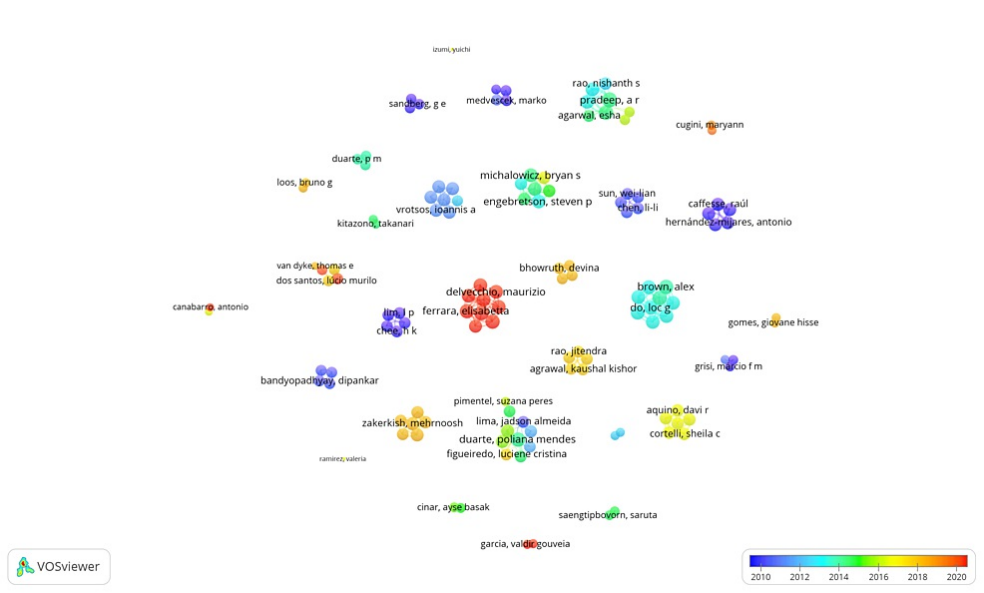
Overlay visualization of the coauthorship analysis of authors. Weight = total link strength values, scores = average publication year, threshold of published clinical trials = 2. The size of the nodes is directly proportional to the total link strength values. The image is created by the author (Namrata Dagli) of this study by using VOSviewer (Leiden, Netherlands: Leiden University).

**Figure 5:**
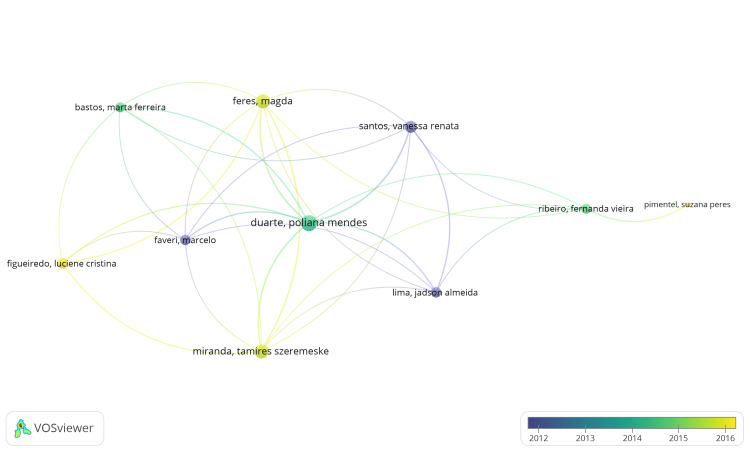
An overlay visualization of the most extensive set of connected items found in the authors' coauthorship analysis. Weight = total link strength values. Each node represents an author, and the connection between nodes represents coauthorship relationships. The image is created by the author (Namrata Dagli) of this study by using VOSviewer (Leiden, Netherlands: Leiden University).

The VOSviewer application identified 1254 authors, among whom 128 authors met the criteria of participation in a minimum of two published clinical trials listed in the PubMed database. Each author's total strength of coauthorship links was calculated using VOSviewer, where the total link strength (TLS) represents the overall strength of connections between authors based on their coauthorship relationships. All 128 authors meeting the criteria were included in the analysis, and an overlay visualization was generated using VOSviewer. The coauthorship analysis revealed that the 128 items were clustered into 30 groups, connected by 277 links with a total link strength of 539. In Figure 4, this network structure is depicted. Notably, author AR Pradeep emerged with the highest total link strength value of 21, indicating significant collaboration within the identified network. The largest cluster comprised 10 items subdivided into three clusters, with 31 links and 47 total link strengths (Figure 5).

Overlay visualization in coauthorship analysis represents the strength of connections between authors through various visual elements overlaid onto a network graph. This visualization method also incorporates temporal analysis. Specifically, when the weight is set to "total link strength," it signifies that the strength of the connection between two authors is determined by the total number of coauthored documents they have collaborated on.

Coauthorship Analysis and Network Visualization of Institutions

**Figure 6:**
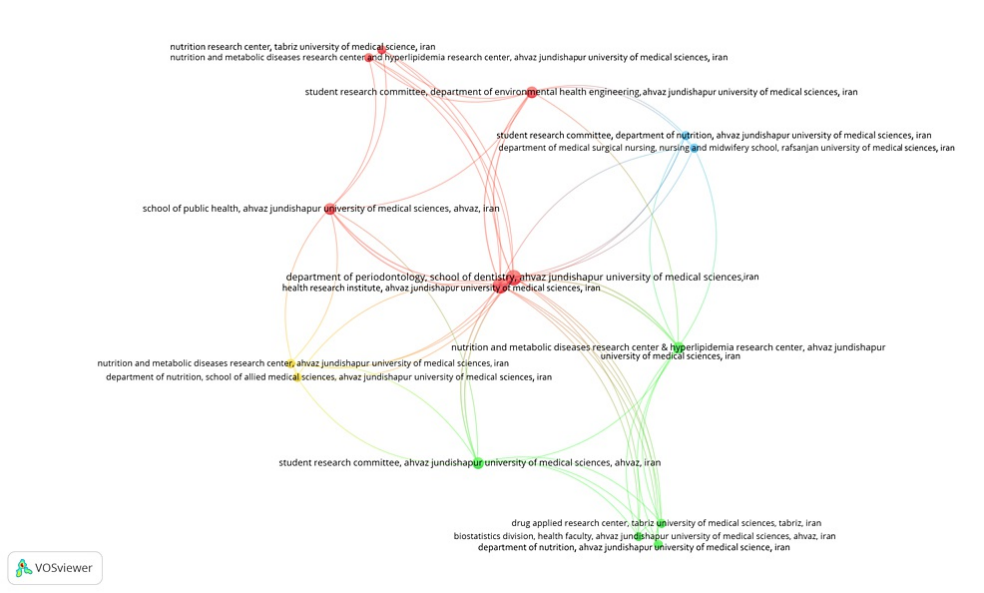
Network visualization of coauthorship analysis of the most extensive set of connected institutions. Weight = total link strength values. Each node represents an institution. Cluster 1 is red, cluster 2 is green, cluster 3 is blue, and cluster 4 is yellow. The image is created by the author (Namrata Dagli) of this study by using VOSviewer (Leiden, Netherlands: Leiden University).

A total of 606 organizations were identified in the analyzed dataset, among which only 28 met the threshold of having published at least two clinical trials. For each of these 28 organizations, the total strength of coauthorship links with other organizations was calculated using the VOSviewer App. These 28 organizations were grouped under 10 clusters, resulting in 42 links and 79 total link strength (TLS). Notably, two departments at Ahvaz Jundishapur University of Medical Sciences in Iran exhibited the highest value of total link strength, with a score of 21. These departments are the Department of Periodontology within the School of Dentistry and the Diabetes Research Center within the Health Research Institute at Ahvaz Jundishapur University of Medical Sciences in Iran which have published four clinical trials each. Furthermore, the most extensive interconnected set consists of 15 organizations spread across four clusters, featuring 55 links and 66 TLS (Figure 6). 

Cooccurrence Analysis of Keywords

**Figure 7:**
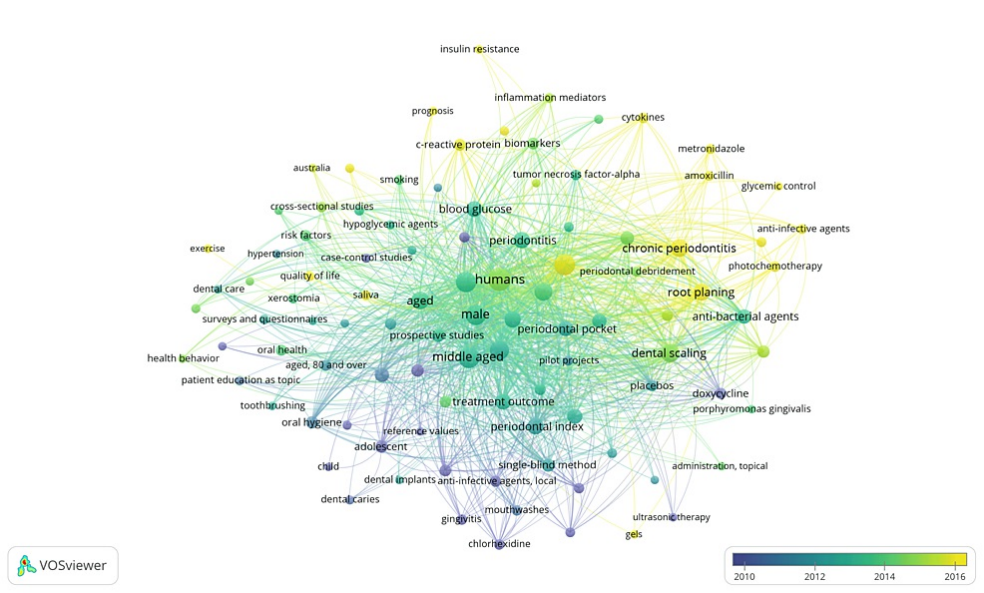
Overlay visualization of cooccurrence analysis of keywords. The image is created by the author (Namrata Dagli) of this study by using VOSviewer (Leiden, Netherlands: Leiden University).

VOSviewer identified a total of 605 MeSH keywords. When the threshold of minimum occurrence was set to five, only 100 keywords met the criteria. For each of these 100 keywords, VOSviewer calculated the total strength of the cooccurrence. The 100 keywords were grouped under six clusters with 2686 links and 16967 TLS (Figure 7). 

Among these keywords, those with the highest total link strength encompass general terms, such as "humans," "middle-aged," "male," "female," and "diabetes mellitus type 2." This highlights the most researched population demographics and diabetes type. Furthermore, the analysis identified subject-specific MeSH keywords with the highest total link strength, indicating a strong association within a particular thematic context. These include "glycated hemoglobin," "dental scaling," "chronic periodontitis," "periodontal index," and "root planning." This suggests a significant focus on specific aspects of diabetes-related oral health, particularly in glycemic control (glycated hemoglobin) and periodontal health (dental scaling, chronic periodontitis, periodontal index, and root planning).

The items in the clusters are mentioned in Table 1. The keywords in cluster 1 suggest a diverse range of research methodologies and areas of investigation in clinical trials concerning the impact of diabetes on oral health. These include epidemiological studies like cohort and case-control studies, intervention studies such as prospective trials and clinical trials, behavioral studies focusing on health behaviors and patient education, and assessments of outcomes like quality of life and severity of illness. Demographic and clinical factors like age, sex, BMI, and comorbidities such as hypertension and cardiovascular diseases are also considered. Overall, the keywords indicate research focused on a holistic understanding of how diabetes influences oral health outcomes and the effectiveness of various interventions and lifestyle factors in managing these issues.

**Table 1:** Keywords in clusters identified in cooccurrence analysis of keywords.

Serial no.	Clusters	Keywords in the clusters
1	Cluster 1 (39 items)	Adult, age factors, aged, aged 80 and over, Australia, body mass index, cardiovascular diseases, case-control studies, chi-square distribution, chronic disease, cohort studies, cross-sectional studies, dental care, dental implants, female, gingival hemorrhage, health behavior, health education, dental, humans, hypertension, hypoglycemic agents, lifestyle, male, middle-aged, oral health, oral hygiene, patient education as a topic, periodontal diseases, prospective studies, quality of life, risk factors, the severity of illness index, sex factors, smoking surveys, questionnaire, tooth loss, toothbrushing, xerostomia
2	Cluster 2 (15 items)	Amoxicillin, antibacterial agents, anti-infective agents, chronic periodontitis, combined modality therapy, dental scaling, type 2 diabetes mellitus, doxycycline, glycated hemoglobin, glycemic control, metronidazole, photochemotherapy, photosensitizing agents, Porphyromonas gingivalis, root planing
3	Cluster 3 (14 items)	Administration topical, alveolar bone loss, double-blind method, follow-up studies, gels, gingival crevicular fluid, gingival recession, β-hydroxy methyl glutaryl-CoA (HMG-CoA), reductase inhibitors, periodontal attachment, periodontal debridement, periodontal index, periodontal pocket, placebos, treatment outcome
4	Cluster 4 (12 items)	Adolescent, child, dental caries, dental plaque, diabetes complications, diabetes mellitus, type 1 diabetes mellitus, gingivitis, periodontitis, reference values, saliva, young adult
5	Cluster 5 (11 items)	Biomarkers, blood glucose, c-reactive protein, cytokines, inflammation, inflammation mediators, insulin resistance, interleukin-6, prognosis, time factors, tumor necrosis factor-alpha
6	Cluster 6 (9 items)	Analysis of variance, local anti-infective agents, chlorhexidine, dental plaque index, mouthwashes, pilot projects, single-blind method, non-parametric statistics, ultrasonic therapy

The keywords in cluster 2 indicate clinical research on the impact of diabetes on oral health, focusing on periodontal diseases and associated complications. Studies explore various interventions such as antibiotics, dental procedures like scaling and root planing, and innovative therapies like photochemotherapy. They aim to understand how glycemic control influences oral health outcomes and may target specific pathogens like *Porphyromonas gingivalis*. Overall, these trials seek to improve management strategies for periodontal diseases in individuals with type 2 diabetes mellitus.

The keywords in cluster 3 indicate a comprehensive focus within clinical trials investigating the impact of diabetes on oral health. Studies encompass various facets, including assessing changes in alveolar bone loss, utilizing rigorous double-blind methods for unbiased evaluation, and conducting follow-up studies to understand long-term outcomes. Interventions often involve topical administration of gels or periodontal debridement to address issues such as gingival recession and periodontal pocket formation. Analysis of gingival crevicular fluid offers insights into inflammatory processes, while medications like reductase inhibitors are explored for their potential benefits. Standardized measures like the periodontal index aid in quantifying disease severity, with placebos used for comparison in treatment evaluations. Overall, the research endeavors to elucidate treatment outcomes of effective interventions for managing oral health complications in individuals with diabetes.

The keywords in cluster 4 suggest that research should focus on oral diseases in younger populations with diabetes, particularly type 1 diabetes. Additionally, studies may focus on establishing reference values for oral health parameters and exploring changes in saliva composition and flow rates associated with diabetes.

The keywords in cluster 5 suggest a research emphasis on comprehending the underlying mechanisms and identifying potential therapeutic targets or prognostic indicators for effectively managing oral health complications in diabetes through meticulously designed clinical trials.

The keywords in cluster 6 indicate research focus on evaluating the efficacy of interventions such as local anti-infective agents and ultrasonic therapy, employing statistical methods like analysis of variance and non-parametric statistics, alongside assessing oral hygiene practices and exploring novel methodologies in clinical trials investigating the impact of diabetes on oral health.

These keywords collectively reflect a variety of research approaches and topics, encompassing investigations into periodontal diseases and their complications in individuals with diabetes, oral health issues in younger populations, especially those with type 1 diabetes, exploration of the underlying mechanisms and therapeutic targets related to diabetes-induced oral health complications, and assessments of interventions and methodologies in clinical trials examining the influence of diabetes on oral health.

Analysis of Topic Trends

The analysis indicates a sustained interest in researching various aspects of type 2 diabetes mellitus, glycated hemoglobin, periodontitis, and therapy for chronic periodontitis over the years. These topics have consistently been prominent in the literature, with repeated emphasis observed across different periods. Before 2013, there was a notable focus on terms such as type 2 diabetes mellitus, glycated hemoglobin, periodontitis, and therapy for chronic periodontitis. This suggests that these topics were already of significant interest to researchers during that period. Between 2013 and 2016, the same terms were repeated, indicating a continued emphasis on these areas of study. This suggests that the relevance of these topics persisted over time. Similarly, between 2016 and 2019, and again between 2020 and 2024, these terms were recurring, highlighting their enduring importance in the research community.

However, the analysis also reveals a shift in the type of therapies being investigated over time. Before 2010, there was a focus on therapies such as doxycycline and local anti-infective agents. There was a transition towards antibacterial agents in 2014, followed by a shift to combined modality therapy in 2015. More recent years have seen research into innovative therapies, such as photosensitizing agents in 2019 and photochemotherapy methods in 2021.

**Figure 8:**
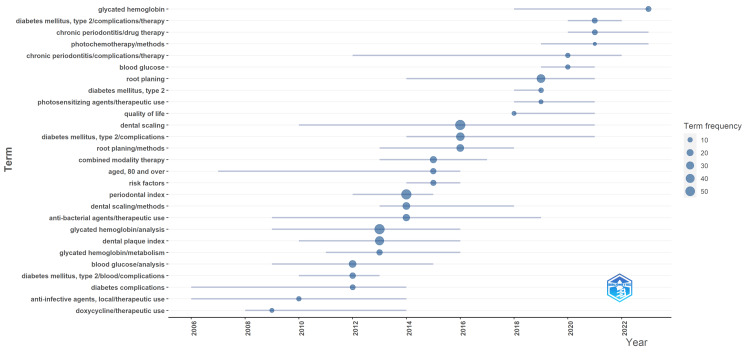
Analysis of topic trends in clinical trials in PubMed on the impact of diabetes on oral health. The image is created by the author (Namrata Dagli) of this study.

Additionally, certain keywords emerged as particularly prominent during specific years. For example, dental scaling was a significant keyword in 2016, indicating a focus on this aspect of periodontal care during that time. Similarly, the periodontal index was prominent in 2014, suggesting a specific focus on periodontal assessment and measurement. Glycated hemoglobin analysis was highlighted in 2013, indicating a particular interest in this aspect of diabetes management during that year. Overall, these findings suggest a persistent interest in researching type 2 diabetes mellitus, glycated hemoglobin, periodontitis, and therapy for chronic periodontitis over time, with a noticeable evolution in the types of therapies being explored and specific areas of focus within each year (Figure 8).

Thematic Evolution

**Figure 9:**
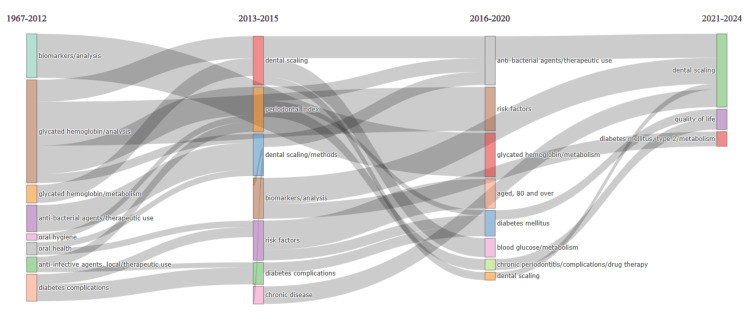
Thematic evolution analysis of keywords used in clinical trials on the impact of diabetes on oral health. The image is created by the author (Namrata Dagli) of this study by using the Biblioshiny App (Naples, Italy: Aria and Cuccurullo).

Figure 9 depicts the most used terms during various periods. Before 2012, the most frequently used terms were biomarker analysis, glycated hemoglobin analysis, glycated hemoglobin metabolism, therapeutic use of antibacterial agents, oral hygiene, oral health, therapeutic use of local anti-infective agents, and diabetes complications. These keywords indicate a research focus on a foundational understanding of diabetes and mitigating its impact on oral health using various anti-infective and antibacterial agents.

The most used keywords during the period spanning from 2013 to 2015 are dental scaling, periodontal index, dental scaling methods, biomarker analysis, risk factors, diabetes complications, and chronic disease. These keywords indicate that the research focuses on the interrelationship between periodontal health and diabetes.

The most commonly used keywords during the period spanning from 2016 to 2020 are therapeutic use of antibacterial agents, risk factors, glycated hemoglobin metabolism, diabetes mellitus, blood glucose metabolism, drug therapy of chronic periodontitis complications, and dental scaling. These keywords indicate the research focus has shifted again to drug therapy with dental scaling for the management of chronic periodontitis complications associated with diabetes mellitus.

The most commonly used keywords during the period from 2021 to 2024 are dental scaling, quality of life, and metabolism of type 2 diabetes mellitus. This suggests a persistent research focus on periodontal health, coupled with a growing consideration of quality of life and metabolic aspects of diabetes. The current study findings suggest a persistent research focus on the complex interactions between diabetes and oral health and their impact on overall well-being over different periods.

Thematic Map

**Figure 10:**
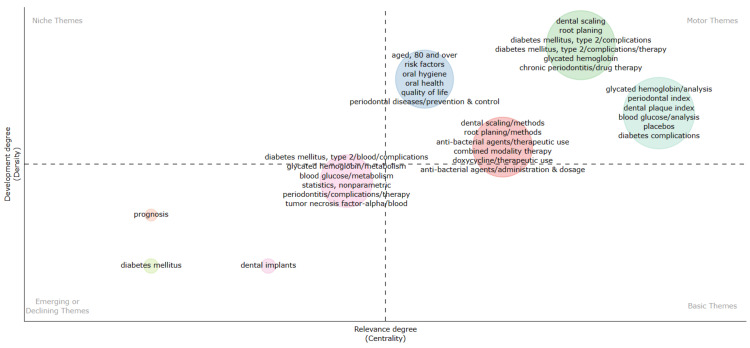
Thematic map (minimum frequency occurrence of keywords per thousand documents=25). The image is created by the author (Namrata Dagli) of this study by using the Biblioshiny App (Naples, Italy: Aria and Cuccurullo).

The thematic map classifies themes into four categories based on their relevance and development levels, illustrated in Figure 10, with motor themes being the most relevant and developed. In our analysis, themes such as dental scaling, root planing, complications and therapy of type 2 diabetes mellitus, glycated hemoglobin analysis, drug therapy for chronic periodontitis, prevention and control of periodontal diseases, oral hygiene, quality of life, periodontal index, dental plaque index, blood glucose analysis, diabetes complications, placebos, combined modality therapy, therapeutic use of antibacterial agents, and therapeutic use of doxycycline are identified as motor themes. Emerging and declining themes denote those gaining or losing importance, respectively. This category includes prognosis, diabetes mellitus, dental implants, complications of type 2 diabetes mellitus, complications of periodontal therapy, tumor necrosis factor-alpha, metabolism of blood glucose, and glycated hemoglobin. Niche themes, which are well-developed but non-relevant, and basic themes, which are relevant but not well-developed, couldn't be identified.

Scientific Production Analysis of Countries

**Figure 11:**
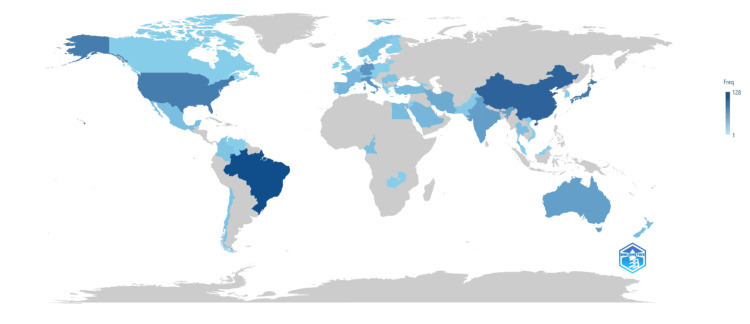
Scientific production of countries. The image is created by the author (Namrata Dagli) of this study.

Figure 11 depicts the scientific production of various countries, ranging from 1 to 128 publications. The intensity of the blue color indicates the number of published clinical trials on the impact of diabetes. Brazil has published the maximum number of clinical trials on the topic (i.e., 128), followed by China with 106 publications. The other countries with more than 50 publications are Japan with 81 publications, the United States with 79 publications, Italy with 55, and Germany with 54.

**Figure 12:**
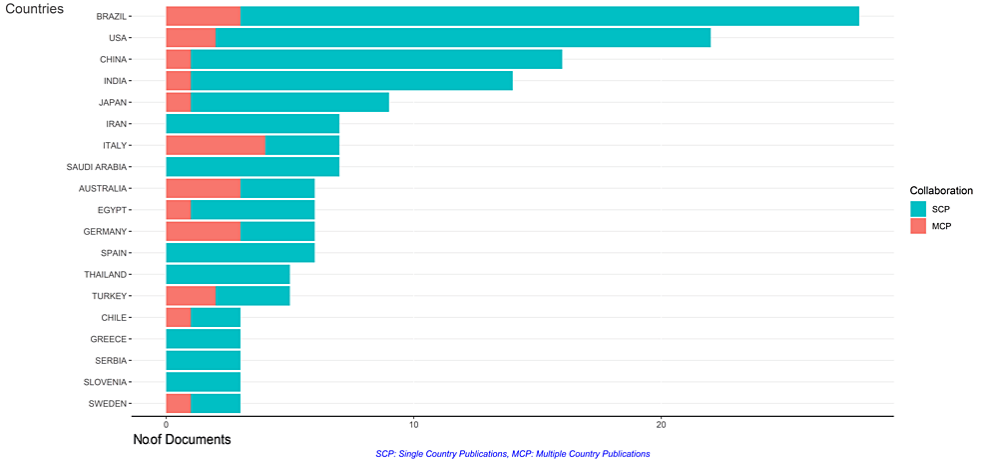
Analysis of countries of corresponding authors. The image is created by the author (Namrata Dagli) of this study.

Analysis of the corresponding authors' countries revealed that most papers are single-country publications. Brazil has produced the maximum number of clinical trials, followed by the United States and China, but most are single-country publications. Italy has published the highest number of multicountry publications, followed by Brazil, Australia, and Germany. Iran, Spain, Thailand, Greece, Serbia, and Slovenia don't show any collaborative efforts, while China, India, Japan, Egypt, Chile, and Sweden show very little collaboration with other countries (Figure 12).

Discussion

The bibliometric analysis provides valuable insights into the research landscape surrounding the impact of diabetes on oral health. A comprehensive understanding of the field emerges by examining publication trends, authorship patterns, keyword cooccurrence cinematic evolution, and country-wise scientific production.

Analysis of publication trends reveals a significant volume of research activity, particularly in recent years, indicated by the high number of publications. This underscores the growing recognition of the importance of understanding the interplay between diabetes and oral health. While there are fluctuations in publication rates over time, there is an overall increasing trend, suggesting sustained interest of researchers in the topic. The diversity of publication types reflects the multidisciplinary nature of research encompassing clinical, epidemiological, and translational investigations and commentary and synthesis of existing literature. These findings underscore the continued scholarly inquiry and collaboration in addressing the complex interplay between diabetes and oral health and advancing clinical practice and public health initiatives in this domain. Coauthorship analysis reveals clusters of authors with varying degrees of collaboration, contributing to disseminating knowledge and expertise across institutions and countries. The clustering of keywords provides insights into the diverse research methodologies, interventions, and outcomes explored in clinical trials. The keywords collectively represent a diverse range of research methodologies and topics, including studies on periodontal diseases and their complications in individuals with diabetes, oral health concerns among younger populations, particularly those with type 1 diabetes, investigations into the underlying mechanisms and therapeutic targets associated with diabetes-induced oral health complications, and evaluations of interventions and methodologies in clinical trials examining the impact of diabetes on oral health. This indicates the focus of clinical trials on diabetes is mainly on periodontal disease, particularly periodontitis. Diabetes can adversely affect oral health by increasing the risk of conditions, such as xerostomia, infections, delayed wound healing, burning mouth syndrome, gingival enlargement, altered taste sensation, dry socket, poor healing after dental procedures, and periodontitis. However, the literature on clinical trials that include these aspects is scarce. The analysis also suggests that all age groups have been included in the clinical trials.

The analysis of topic trends illustrates the shift in research focus over time, with a consistent emphasis on topics such as glycated hemoglobin, periodontitis, and therapy for chronic periodontitis. While certain themes remain prominent throughout different periods, such as type 2 diabetes mellitus, glycated hemoglobin, periodontitis, and therapy for chronic periodontitis, there are shifts in the kinds of therapies investigated, reflecting advancements in treatment modalities and changing research priorities. Thematic map suggests that tumor necrosis factor-alpha and dental implants are emerging topics. Tumor necrosis factor-alpha (TNF-α) plays a significant role in the pathophysiology of both diabetes and oral health, contributing to insulin resistance, inflammation, and tissue destruction, thus linking diabetes to periodontal diseases and other oral health complications [19].

Brazil emerges as a leading contributor to research on the impact of diabetes on oral health, followed closely by China, Japan, the United States, Italy, and Germany. While single-country publications are prevalent, there are also instances of international collaboration, particularly in multicountry publications. This indicates a global interest in addressing the complex challenges of diabetes-related oral health issues.

**Figure 13:**
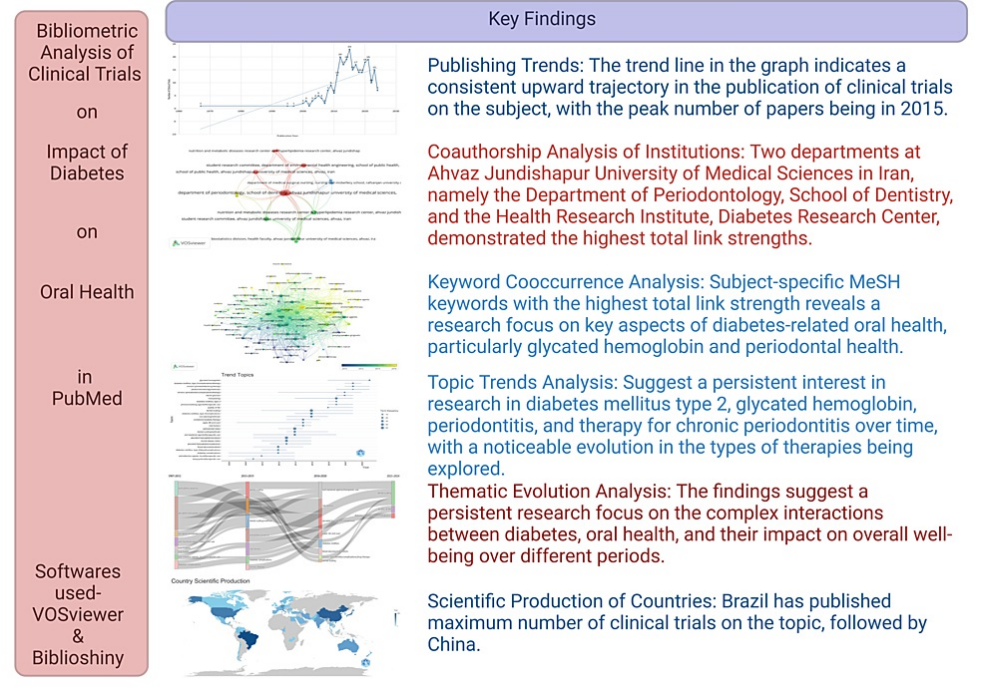
Key findings of bibliometric analysis on the impact of diabetes on oral health. The image is created by the author (Namrata Dagli) of this study using the premium version of BioRender (ON, Canada: Science Suite Inc.).

To the best of our knowledge, no bibliometric analysis on this topic has been done till now. However, few bibliometric studies that explored the literature regarding the relationship between diabetes focused on any one aspect related to oral health, such as periodontal diseases and oral implants [20-23]. Our bibliometric analysis sheds light on the robust research landscape concerning the impact of diabetes on oral health, indicating a growing recognition of the importance of this interplay. Most of the articles are focused on periodontal disease. Other oral health conditions related to diabetes need the attention of researchers. This insight can inform healthcare professionals, policymakers, and educators about the need for continued attention to diabetes-related oral health issues. Additionally, identifying publication trends and thematic evolution guides researchers, highlighting areas of sustained interest and emerging topics for further investigation. According to our thematic analysis, the TNF-α, and dental implants are emerging topics that are gaining more attention. The multidisciplinary nature of the research, as evidenced by diverse publication types and collaborative authorship patterns, underscores the importance of interdisciplinary collaboration in addressing complex health challenges. Moreover, the prominence of certain countries, such as Brazil, China, and the United States, suggests opportunities for international collaboration to enhance knowledge exchange and research capacity in this field. Overall, the bibliometric analysis provides a comprehensive understanding of the current research landscape and offers practical insights for advancing public health initiatives and future research directions in the domain of diabetes-related oral health. We have summarized the key findings in Figure 13.

Although attempts were made to achieve thorough coverage, it's essential to recognize the limitations of utilizing the PubMed database and the selected search approach in this bibliometric analysis. Moreover, by only including publications in English, there is a risk of introducing language bias, potentially overlooking pertinent studies published in other languages. Additionally, while bibliometric analysis offers valuable insights into publication trends and thematic patterns, it does not assess the quality or reliability of individual studies incorporated within the analysis.

Future study recommendations

The findings from the bibliometric analysis on the impact of diabetes on oral health suggest several avenues for future research. Longitudinal studies could provide valuable insights into the progression of oral health complications in individuals with diabetes, enabling researchers to assess the efficacy of interventions over time. Moreover, there is a need for interventional trials focusing on novel therapies and personalized treatment approaches to improve oral health outcomes in diabetic patients. Collaboration between dental professionals, endocrinologists, and other specialists could facilitate multidisciplinary research to understand better the complex interplay between oral health, systemic health, and lifestyle factors. Additionally, conducting health economics studies to evaluate the economic burden of oral health complications and assess the treatments' cost-effectiveness could inform healthcare policy decisions. Future research should also prioritize patient-centered outcomes and incorporate qualitative research methods to capture the lived experiences and preferences of individuals with diabetes and oral health issues. However, it's important to acknowledge the limitations of bibliometric analysis, including publication bias, language bias, and database limitations, which may impact the comprehensiveness and interpretation of the findings. Future studies should address these limitations to ensure robust and reliable research outcomes in this vital area of healthcare.

## Conclusions

The bibliometric analysis conducted on the impact of diabetes on oral health reveals a comprehensive landscape of research trends, publication patterns, authorship collaborations, thematic evolution, and international scientific production. The distribution of publication types indicates a diverse range of scholarly contributions, including reviews, case reports, clinical trials, and observational studies, highlighting the multifaceted approach to understanding this complex interplay. The Department of Periodontology within the School of Dentistry and the Diabetes Research Center within the Health Research Institute at Ahvaz Jundishapur University of Medical Sciences in Iran are significant collaborators within the research community. The study identifies persistent research focus on type 2 diabetes mellitus, glycated hemoglobin, periodontitis, and therapy for chronic periodontitis. However, there's a shift in the kinds of therapies investigated. The thematic map indicates that "dental implant" and "tumor necrosis factor-alpha" are emerging concepts in the field, recently gaining increasing attention and relevance. In addition, the analysis highlights the research gap and the need for more clinical trials on other aspects of oral health beyond periodontitis. Furthermore, the analysis highlights the involvement of numerous authors and institutions in collaborative efforts, albeit with varying degrees of international collaboration. Brazil is a leading contributor to scientific publications in this field, followed by China, Japan, the United States, Italy, and Germany. Continued interdisciplinary collaboration and international cooperation are essential for advancing knowledge and improving patient care in this critical area of healthcare.
